# A System of Medicine

**Published:** 1897-09

**Authors:** 


**A System of Medicine.** By Many Writers. Edited by THOMAS CLIFFORD ALLBUTT, M.D., F.R.S. Vol. II. Pp. xiv., 1176. London: Macmillan and Co., Limited. 1897.

The topics considered in this volume are the various infective diseases not previously described, and the first article is by Dr. Sidney Martin on Tuberculosis, which is described as "a chronic febrile disease, produced by the bacillus tuberculosis." Everything is circled round the bacillus. When this does not enter the body no tuberculosis can be manifested, and other diseases "only dispose to the reception and implantation of the microorganism from the environment." There is no longer any hesitation in classing the malady amongst the infective diseases, and we no longer hear any doubts as to the existence of a contagion, or as to the contagiousness of the disease. "Tuberculosis is an infection; it is due, that is, to a virus which is introduced into the body; it cannot arise *de novo* in the body," hence it follows that the demonstration of the bacillus is the first important question: its characters, its mode of growth, its cultivation, its chemical products, its pathological effects, the modes of infection in animals and man, the sources of infection, and the spread of the disease in the body after infection,—these are the principal topics dealt with. It is clearly laid down that the bacillus is a parasite, and not a saprophyte; that it has no independent existence outside the body, that it exists only in tuberculous lesions and their products, and hence in the human being tuberculous infection is not of universal distribution, but is limited to places inhabited by consumptives, and in cattle it is a disease of the stall. The sources of infection are all under control. Precautionary measures are available against its invasion and its spread, and hence the arguments in favour of notification summed up by Dr. Niven (*Medical Chronicle*, April, 1897,) are in all respects stronger than those used on behalf of the notification of any infectious disease, "both as regards our knowledge, even at the present, of the modes of infection, and as regards the good which we may hope to effect." The article concludes with the remark that "there is no specific treatment for tuberculosis." There are, however, indications of light on the horizon, and we trust that the second edition of this book will give us a more hopeful outlook for the victims of this widely-spread infection. Dr. Phineas Abraham has also to confess that no specific drug has yet been discovered for leprosy; but as regards the other chronic infective disease, actinomycosis, Dr. Theodore Dyke Acland remarks that "the organism, fortunately, has a low vitality, and is readily affected by iodoform, corrosive sublimate, and other antiseptics." May we not expect that leprosy and tuberculosis may be even more effectually influenced by some antitoxin or antiseptic than appears yet to be within our reach?

The chapter on the diseases of uncertain bacteriology which are not endemic are written by Dr. Dawson Williams, Dr. Caiger, Dr. John MacCombie, Dr. Eustace Smith, and Mr. Jonathan Hutchinson. Those on the topical or endemic diseases are by well-known authorities on the different subjects.

The diseases due to protozoa are described by Dr. William Osler, Dr. S. Monckton Copeman, and Dr. Henri A. Lafleur, whose monograph on Amœbic Dysentery gives us everything known on this disease.

The following chapters on Intoxications include a variety of subjects by various authors: poisoning by food (ptomaine poisoning), by Dr. Sidney Martin; grain poisoning, mushroom poisoning, opium poisoning and other intoxications, by Dr. Clifford Allbutt; snake poison and snake-bite, by Mr. Charles James Martin and Dr. Calmette; alcoholism, by Dr. Rolleston; and metallic and some other forms of poisoning, including poisonous trades, by Dr. Thomas Oliver. These somewhat obscure and indefinite subjects are dealt with in a very complete and instructive manner, and will well repay a careful perusal.

A chapter on Psorospermosis, and the usual internal parasites, completes the volume. We notice that chloroform is not mentioned amongst the useful anthelmintics, whereas we have found it to be one of the most efficient of them all.

